# Fusarium oxysporum f.sp. radicis-lycopersici induces distinct transcriptome reprogramming in resistant and susceptible isogenic tomato lines

**DOI:** 10.1186/s12870-016-0740-5

**Published:** 2016-02-27

**Authors:** Daniele Manzo, Francesca Ferriello, Gerardo Puopolo, Astolfo Zoina, Daniela D’Esposito, Luca Tardella, Alberto Ferrarini, Maria Raffaella Ercolano

**Affiliations:** Department of Agriculture Sciences, University of Naples ‘Federico II’, Via Università, 100, 80055 Portici, Italy; Current address: Sustainable Agro-Ecosystems and Bioresources Department – IASMA Research and Innovation Center – Fondazione Edmund Mach, S. Michele all’Adige, Trento, Italy; Department of Statistical Sciences, University of Rome ‘La Sapienza’, Rome, Italy; Dipartimento di Biotecnologie, Università degli Studi di Verona, Strada le Grazie, Verona, Italy

**Keywords:** *Solanum lycopersicum*, FORL resistance, Necrotrophic pathogen, Transcriptomic, Callose deposition, Dehydration-induced protein, Oxidative burst, Necrosis reaction

## Abstract

**Background:**

*Fusarium oxysporum f.sp. radicis-lycopersici* (FORL) is one of the most destructive necrotrophic pathogens affecting tomato crops, causing considerable field and greenhouse yield losses. Despite such major economic impact, little is known about the molecular mechanisms regulating *Fusarium oxysporum f.sp. radicis-lycopersici* resistance in tomato.

**Results:**

A transcriptomic experiment was carried out in order to investigate the main mechanisms of FORL response in resistant and susceptible isogenic tomato lines. Microarray analysis at 15 DPI (days post inoculum) revealed a distinct gene expression pattern between the two genotypes in the *inoculated vs non-inoculated* conditions. A model of plant response both for compatible and incompatible reactions was proposed. In particular, in the incompatible interaction an activation of defense genes related to secondary metabolite production and tryptophan metabolism was observed. Moreover, maintenance of the cell osmotic potential after the FORL challenging was mediated by a dehydration-induced protein. As for the compatible interaction, activation of an oxidative burst mediated by peroxidases and a cytochrome monooxygenase induced cell degeneration and necrosis.

**Conclusions:**

Our work allowed comprehensive understanding of the molecular basis of the tomato-FORL interaction. The result obtained emphasizes a different transcriptional reaction between the resistant and the susceptible genotype to the FORL challenge. Our findings could lead to the improvement in disease control strategies.

**Electronic supplementary material:**

The online version of this article (doi:10.1186/s12870-016-0740-5) contains supplementary material, which is available to authorized users.

## Background

*Fusarium oxysporum* f.sp. *radicis-lycopersici* (FORL) is a necrotrophic pathogen, causal agent of tomato crown and root rot, a disease of worldwide economic importance in commercial tomato. The disease results in severe losses in the greenhouse, field crops and hydroponic cultures [1]. Although various methods have been employed to control this pathogen, the use of resistant cultivars is the most acceptable and economic system of control [2]. In tomato the *Frl* gene, which confers partial resistance to FORL, was mapped on the long arm of chromosome 9 in linkage drag with the *Tm-2* locus [3]. To date, little information on genes involved in resistance to FORL has been released [4]. Genomic-based approaches have proved to be very useful to identify genes involved in plant-pathogen interactions [5]. In wheat, a microarray-based approach revealed a distinctive transcriptome pattern for each plant organ (glume, lemma, palea, anther, ovary and rachis) in response to *F. graminearum* infection [6]. Transcriptome analysis also proved very useful in identifying genes involved in *Fusarium* head blight (FHB) resistance in a Chinese wheat landrace [7]. Transcriptome profiling of watermelon during its incompatible interactions with *F. oxysporum* f.sp. *niveum* (FON) showed that transporter proteins might contribute to the development of wilt symptoms [8]. Increased expression of defense-related genes were also observed in tomato plants infected by *F. oxysporum* f.sp. *lycopersici* [9].

Beyond plant-microbe interactions, transcriptomic approaches have been widely used in discovering pathogen colonization habits. For this purpose, Carapito and colleagues [10] reported a genome-wide transcriptomic analysis of *F. graminearum*, providing new insights of the biology of this pathogen in the presence of different polysaccharide sources. An NGS (Next Generation Sequencing) approach helped to understand the molecular underpinning of pathogenicity in *F. oxysporum* f.sp. *cubense* (FOC), a causal agent of banana vascular wilt disease [11]. Indeed, transcriptome analysis was very useful in revealing the pattern of pathogen activities and molecular repertoires available for defense responses, allowing dissection of the molecular basis of plant-pathogen interaction.

Despite the importance of the disease caused by FORL, little is known about tomato genome reprogramming during the onset of the disease. More detailed knowledge on the interaction between tomato and this soil-borne fungus could lead to the discovery of more efficient ways to control the disease. The aim of the present study was to investigate transcriptional changes in resistant (*Momor*) and susceptible (*Monalbo*) isogenic tomato lines after infection by FORL and to compare results between compatible and incompatible interactions. Moreover, in order to shed more light on this kind of interaction we attempted to produce a model of plant response during both compatible and incompatible reactions based on the study of interconnected pathways evidenced in our study.

## Methods

### Plants and the fungal strain used in the experiments

The susceptible tomato (*Solanum lycopersicum*) variety *Marmande* was used for initial pathogenicity tests; tomato isogenic varieties *Monalbo* and *Momor*, that have the same *Moneymaker* genetic background except for the *Frl* gene [12], respectively susceptible and resistant to FORL, were used for transcriptional experiments. Tomato varieties, used in our experiments, came from germplasm collection of the Plant Genetics and Biotechnologies section - Department of Agricultural Sciences- University of Naples Federico II. The FORL strain used was For-l F55 NA isolated from a naturally infected tomato plant grown in Battipaglia (Italy) in 2007. The strain For-l F55 NA was routinely maintained in Petri dishes containing Potato Dextrose Agar (PDA; Oxoid) at 24 °C and it was long-term stored at −80 °C in glycerol (20 %).

### Fungal infection assay and plant infection

For-l F55NA fresh conidia were collected from sporulating colonies grown for 14 days on PDA at 24 °C. Petri dishes were flooded with 5 ml of sterile distilled water (SDW) and conidia were scraped using sterile spatulas and transferred in sterile 50 ml tubes. The conidia suspensions of For-l F55NA were then adjusted to a final concentration of 1 × 10^6^conidia/mL by counting with a hemocytometer under a light microscope. *Marmande* plantlets were first grown in sterile peat until the first-leaf stage, then uprooted and dipped for 30 min in a 1 × 10^6^ conidia/ml suspension. Inoculated plantlets were then transferred into sterile sand pots and grown in a greenhouse for 21 days. Plantlets were visually evaluated after 21 days, assessing symptoms according to the following disease index scale: 0) no symptoms; 1) moderate brown lesions on secondary roots and taproot; 2) severe rot on taproot and plant crown; 3) dead or almost dead plantlets. *Monalbo* and *Momor* seedlings were grown in sterile peat until the third-leaf stage, then removed from pots containing peat, and roots were gently washed in order to remove peat debris. Plantlets were then inoculated with For-l F55NA by dipping roots in conidia suspension for 30 min. Plants dipped for 30 min in distilled water were used as controls. Subsequently, the plantlets were transferred to pots containing sterile sand and placed in a growth chamber (22 °C/14 h light, 16 °C/10 h dark). A volume of 5 ml of Hoagland solution [13] was supplied daily to the plantlets during the trials. Two weeks after treatment, plantlets were taken from the pot and the occurrence of tomato crown and root rot were visually scored at 10, 15 and 21 days post-inoculum (DPI), according to the above-mentioned disease index scale. To further confirm the inoculation by For-l F55 NA strain, the fungus was re-isolated from all the tissues of the infected plantlets that showed a disease index scale higher than 1. Tomato plantlets were uprooted and washed under running water; then stem sections were put on Potato Dextrose Agar plates for in vitro growth.

### Sample collection and mRNA isolation

Infected and uninfected root samples of *Momor* and *Monalbo* genotypes were collected at 0 DPI, 7 DPI, 15 DPI and 21 DPI in order to analyze gene expression changes after fungal treatment. For each treatment, 30 plants were employed and all samples were collected in three independently repeated experiments. Roots were removed from plantlets, weighed and immediately frozen in liquid nitrogen and stored at −80 °C. Root total RNA was isolated from the powdered collected samples using the RNeasy Plant Kit (Qiagen) and then treated with DNase I in order to remove any contaminating genomic DNA, following manufacturer’s instructions. RNA integrity was evaluated using the Agilent 2100 Bioanalyzer (Agilent Technologies).

### Chip design and microarray hybridization

Transcriptome analysis was performed on a 90 K TomatArray1.0 microarray synthesized using the Combimatrix platform [http://www.combimatrix.com] at the Plant Functional Genomics Center of the University of Verona. Microarray analysis was used to investigate tomato gene expression profiles 15 days after infection with FORL, comparing it with the profile of uninfected controls. The chip carried 25,789 non-redundant probes (23,282 unique probes and 2507 probes with more than one target) randomly distributed in triplicate across the array. The source of sequence information included tentative consensus sequences (TCs) derived from the DFCI Tomato Gene IndexRelease 12.0 and expressed sequence tags. Total RNA (2 μg) was amplified to obtain antisense RNA (aRNA) using the SuperScript Indirect RNA Amplification System Kit (Invitrogen). aRNA labeling was performed by incorporating Alexa Fluor 647 Reactive Dye. NanoDrop™ 1000 (Thermo Scientific) was used to check the quantity and quality of both RNA and labeled aRNA of each replica. Two biological replicates were employed for conducting further experiments since few samples of failed control analysis. Labeled aRNA was hybridized to the array according to the manufacturer’s recommendations [http://www.combimatrix.com]. Pre-hybridization, hybridization, washing and imaging were performed according to the manufacture’s protocols. The array was scanned with a Perkin Elmer Scan Array 4000XL (software ScanArray Express Microarray Analysis System v4.0).

### Data analysis

Scanned Combimatrix arrays were analyzed using Bioconductor packages [14]. Arrays were normalized using quantile normalization and expression estimates were compiled by applying the empirical Bayes approach [15]. Differentially expressed probe sets were identified using the R software (R Core Team 2013) and the limma package. Two biological replicates were employed to assess differential expression of each inoculated and non-inoculated genotype to compare the different experimental conditions (inoculated vs non-inoculated) using a linear model for microarray [16]. In our work technical replicates with independently labeled aliquots were up to four for a single RNA sample, non-redundant probes were distributed at least in triplicate across the array and statistical analysis was performed using strictly parameters, avoiding confounding factors. Significance of differential expression analysis was assessed, taking account of the multiple testing setting and controlling the False Discovery Rate (FDR) at FDR = 0.05. All microarray expression data are available at the NCBI’s GEO dataset under the series ID entry GSE71393.

### Annotation gene chip

An in-house pipeline was used to annotate tomato tentative consensus sequences (TCs) used as microarray probes. Tomato genes were identified by mapping TC sequences to the tomato CDS sequence using BlastN (E-value 1e-3). The latest version of the tomato gff3 annotation files was parsed to extract the CDS sequences of gene probes. Blast2GO pipeline (http://blast2go.bioinfo.cipf.es/), with an expectation value threshold of 1e-6 in BlastP analysis, was used to provide automatic high-throughput annotation, gene ontology mapping and categorization of tomato protein identified. Blast2GO was also used for the GO term enrichment analysis based on Fisher’s Exact Test and corrected for multiple testing using an FDR cut-off value of 0.05. The Sol Genomics (www.solgenomics.net) database was useful to find more information on annotated genes, while SolCyc (http://solcyc.solgenomics.net/) was used to obtain detailed information on pathways and biochemical reactions involved in the tomato-FORL interaction. For further reconstructions of pathways involved in the reaction, KEGG database (http://www.genome.jp/kegg/) was interrogated to find enzymes involved in the incompatible and compatible interactions.

### RT qPCR assay

Three qPCR assays were carried out: 1) assay to monitor the activation of reporter genes in *Marmande* at 21 DPI; 2) assay to monitor the FORL disease time-course in *Momor* and *Monalbo* genotypes at 0 DPI, 15 DPI, 21 DPI; 3) assay on *Momor* and *Monalbo* at 0, 7 and 15 DPI to validate microarray results. All qPCR assays were performed according to the Minimum Information for Publication of Quantitative Real-Time PCR Experiments guidelines (MIQE) [17] and are described as follows. All PCR reactions were performed in triplicates using SensiFast SYBR Hi-Rox Kit (Bioline) on Rotor-Gene 6000™ (CorbettResearch, CYBELES, Thailand) according to the manufactures instructions. A total of 1 μg of the extracted mRNA was used to synthesize first-strand cDNA by using SuperScript® III Reverse Transcriptase Kit (Life Technologies) following the manufacturer’s instructions. Reactions were set up in a final volume of 13 μl containing: 4.5 μl (1:20 diluted) cDNA template, 6.25 μl SensiFast SYBR Hi-Rox 2x, 4.28 μM of primer pair mix and water to make up the total volume. For each primer pair a negative no template control was included using autoclaved double distilled water to replace the cDNA. All samples were normalized to *actin* as reference gene [18, 19], and specific primers for the assays were designed using Primer3 (http://primer3.ut.ee/). All primer sequences are displayed in Additional file 1: Table S1 and the final amplified product size was around 100 bp. Amplification conditions were 40 cycles of 95° for 15 s (denaturation) followed by 60° for 1 s and 72° for 20s (annealing and extension). Data analysis was performed with the RotorGene6000™ Software 1.7 using non-inoculated samples as calibrators and the ΔΔCT method (Livak and Schmittgen, 2001) was performed to analyze expression data.

## Results

### Study of the disease time-course

In order to investigate tomato-FORL interaction we performed an experiment to assess disease evolution in the susceptible cultivar *Marmande*. After 10 DPI few brown lesions (disease index scale 0–1) were observed on secondary roots, at 15 DPI more pronounced rot on taproots and plant crown were evidenced (disease index scale 1–2) and at 21 DPI severe rot on taproot and plant crown (disease index scale 2–3) were visible. A Real-time quantitative polymerase chain reaction (qPCR) of genes playing a key role in pathogen response such as Phenylalanine ammonia-lyase (PAL), Catalase, Receptor-like protein kinase (RLK) 4 Serine/Threonine and Beta-glucosidase was performed in order to monitor the FORL response induced in infected and non-infected *Marmande* root samples (Additional file 2: Figure S1). Such genes were chosen because their expression provide indirect evidence of defense response activation against environmental stress stimuli [20, 21]. PAL, Beta-glucosidase and RLK4 Serine/Threonine genes were up-regulated in the infected samples, while the Catalase gene was down-regulated. These results confirmed a differential response between infected and non-infected samples. Subsequently, Catalase and Beta-glucosidase were assessed in the two isogenic genotypes for resistance to FORL (*Momor* and *Monalbo*) at 0 DPI, 15 DPI, 21 DPI (data not shown). In the resistant genotype *Momor*, the Catalase gene, proved up-regulated at any time recorded after inoculation, while in the susceptible genotype *Monalbo*, its expression decreases from 15 to 21 DPI. Beta-glucosidase gene expression is down-regulated at 0 DPI in the resistant genotype and then up-regulated at 15 and 21 DPI. By contrast, in the susceptible genotype, its expression is up-regulated at 0 DPI, increases at 15 DPI and then dramatically decreases at 21 DPI. These observations were used to establish the time for collecting samples for microarray analysis at 15 DPI since at this time point a gene expression switching was detected between the two isogenic lines.

### Genome-wide transcriptional analysis

Microarray transcriptional profiles of a resistant and a susceptible tomato genotypes were used to explore tomato-FORL pathogen interaction at 15 DPI. Four different experiments were carried out in order to make all the possible comparisons among resistance/susceptibility responses (Additional file 1: Table S2). In the first experiment we compared all the transcripts activated or inhibited in the resistant, inoculated and non-inoculated *Momor* genotype (incompatible interaction); in the second experiment we compared the inoculated versus non-inoculated *Monalbo* susceptible genotype in order to explore all the transcripts activated during the susceptible reaction (compatible interaction). In the third experiment the transcriptional changes between the susceptible and resistant genotypes were highlighted (compatible versus incompatible interaction); in the fourth and last experiment we monitored the response in susceptible and resistant non-inoculated samples (control reaction). In the control reaction a very small number of differentially expressed genes was evidenced; among them a LRR receptor (Solyc01g009690.1.1), a Heat shock protein (Solyc09g010630.2.1) and a Universal stress protein (Solyc09g011670.2.1), confirming that the two analyzed genotypes are isogenic.

**Fig. 1:**
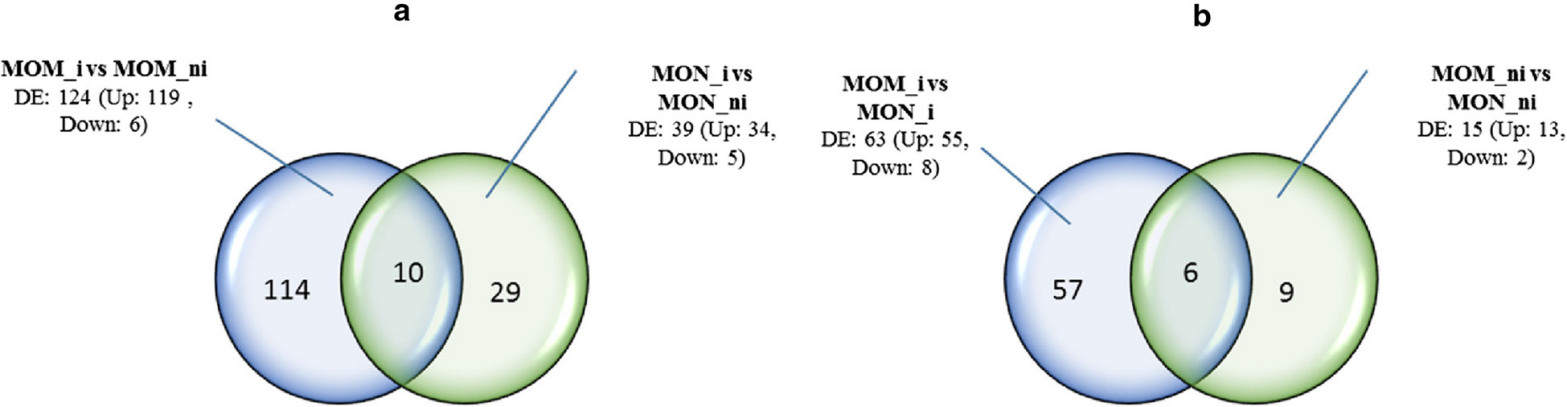
Differentially expressed gene analysis. Venn Diagrams showing the number of unique and overlapping DE genes in the four microarray experiments after 15 DPI (days post inoculum). **a**) MOM i vs MOM ni (incompatible interaction); MON i vs MON ni (compatible interaction). **b**) MOM i vs MON i (compatible vs incompatible interaction); MOM ni vs MON ni (control reaction). MOM_i = *Momor* inoculated; MOM_ni = *Momor* non-inoculated; MON_i = *Monalbo* inoculated; MON_ni = *Monalbo* non-inoculated

Transcriptional responses of resistant and susceptible tomato plantlets, inoculated with FORL, were evaluated by querying 15,734 tomato genes. In the incompatible interaction 124 differentially expressed (DE) genes were observed, while in the compatible interaction 39 DE genes were observed. In particular, 119 genes (about 90 %) were up-regulated in the incompatible interaction, indicating considerable gene activation during the infection process. As for the compatible interaction, 34 genes were up-regulated. In the incompatible versus compatible interaction we observed 63 differentially expressed genes, 55 of which were up-regulated while just 8 were down-regulated. In the first two comparisons, few up-regulated overlapping genes (10) were observed (Fig. 1a), while in the other two comparisons just six overlapping genes were evidenced (Fig. 1b). Comparing gene expression among the four experiments, there were more up-regulated than down-regulated genes, suggesting that genome reprogramming after FORL infection induced high gene activation.

In the incompatible interaction, several genes involved in ethylene biosynthesis were up-regulated, including a putative 1-aminocyclopropane-1-carboxylate (Solyc12g006380.1.1) and an AP2-like ethylene-responsive transcription factor (Solyc03g044300.2.1). A GID1-like gibberellin receptor (Solyc01g098390.2.1) involved in gibberellin signaling components and several genes encoding calcium-dependent proteins like calmodulins were also up-regulated (Solyc02g079040.2.1; Solyc11g071740.1.1; Solyc08g014280.2.1; Solyc01g068460.2.1). Moreover, several up-regulated receptor genes involved in resistance response, including CC-NBS-LRR (Solyc04g015210.2.1 and Solyc04g007050.2.1) and LRR-repeat proteins (Solyc07g066240.2.1) were evidenced. Interestingly, a dehydration-induced protein and a Cytochrome p450 protein was detected during this interaction (respectively Solyc09g092640.2.1 and Solyc12g099390.1.1). The CYP83B1 monooxygenase (Solyc09g092640.2.1) is an enzyme involved in the glucosinolate biosynthesis, tryptophan metabolism and biosynthesis of other secondary metabolites. Moreover, using the Blast2GO tool, some DE genes were assigned to KEGG maps of arginine and proline metabolism (Solyc04g014510.2.1 Glutamine synthetase), glutathione metabolism (Solyc05g006750.2.1 Glutathione S-Transferase), indolic alkaloids pathway (Solyc07g055740.1.1 Strictosidine synthase-like) and phenylpropanoids and lignin biosynthesis (Solyc12g094520.1.1 4-coumarate:CoA ligase) and will be discussed further.

In the compatible interaction, evaluated by comparing the transcriptome of inoculated and non-inoculated susceptible genotype, several up-regulated genes were evidenced. Interestingly, a high activation of genes involved in the fatty acid (and Jasmonate) biosynthesis, including an Omega-6 fatty acid desaturase (Solyc04g040130.1.1), and a Jasmonate ZIM-domain protein (Solyc12g009220.1.1) were observed. An up-regulated 1-aminocyclopropane-1-carboxylate (Solyc12g006380.1.1) gene involved in ethylene biosynthesis and ethylene-responsive transcription factor (Solyc02g077370.1.1) and a down-regulated LRR receptor-like serine/threonine (Solyc01g009690.1.1) were identified. A cytochrome p450 protein (Solyc10g080840.1.1), acting on a wide range of substrates, was also up-regulated during compatible interaction. Up-regulated genes involved in purine metabolism (Solyc11g065930.1.1 Xanthine dehydrogenase/oxidase) and phenylalanine metabolism (Solyc03g025380.2.1 Peroxidase; Solyc04g071890.2.1 Peroxidase 4) were also detected in this comparison.

Comparing directly the dataset of compatible and incompatible genotypes, several over-expressed pathogenesis related (PR) proteins were evidenced in the susceptible genotype, including PR-2 (Beta 1-3-glucanase, Solyc10g079860.1.1 and Solyc01g008620.2.1), PR-3 (Chitinase, Solyc07g009510.1.1), PR-11 (Acidic Chitinase, Solyc05g050130.2.1), PR-6 (Kunitz-type proteinase inhibitor, Solyc03g098710.1.1 – Proteinase inhibitor II, Solyc03g020060.2.1 – Proteinase inhibitor, Solyc11g021060.1.1) and PR-10 (PR-10 related norcoclaurine synthase-like protein, Solyc07g005380.2.1 and pathogenesis-related protein 4B, Solyc01g097240.2.1).

**Fig. 2:**
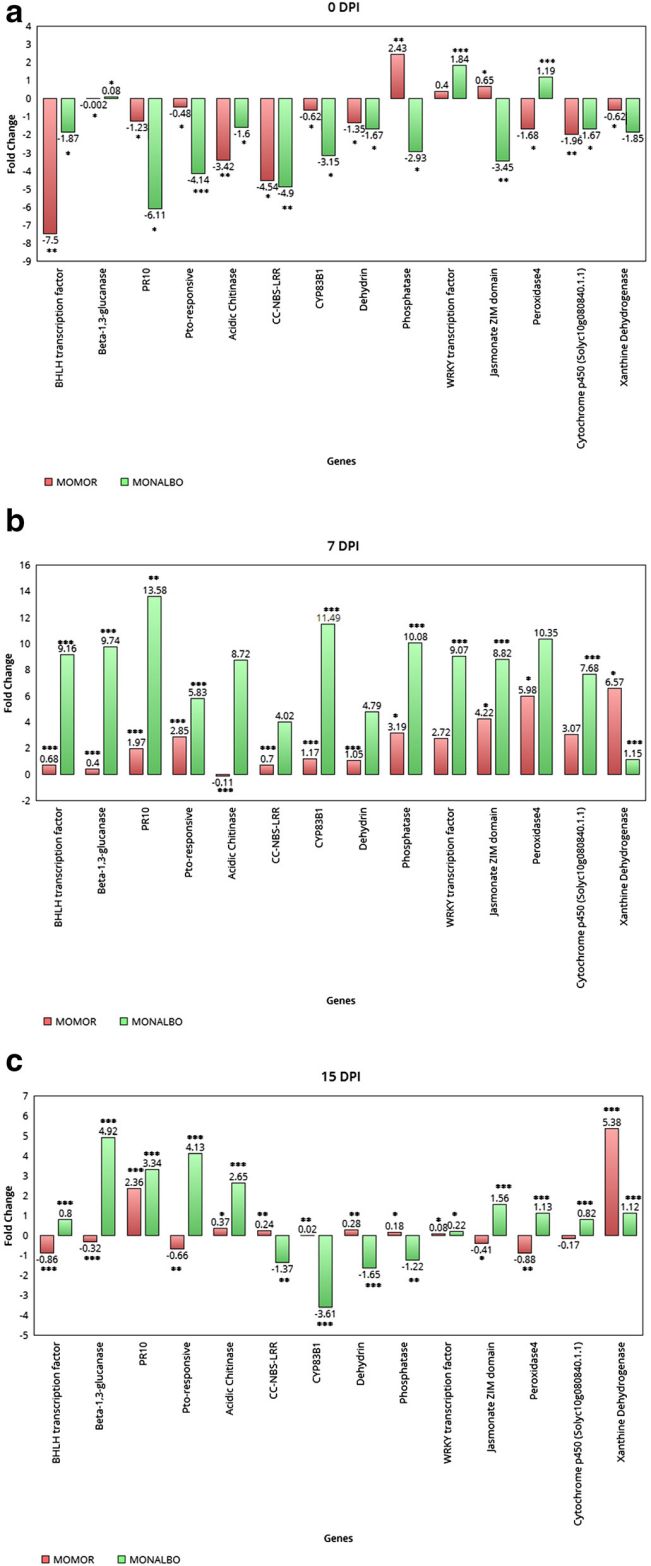
qPCR gene expression profiling. qPCR assay of 14 target genes identified in the tomato-FORL interaction. At 0 DPI (panel **a**), 7 DPI (panel **b**) and 15 DPI (panel **c**). Bars indicate real-time expression measurements (Fold Change) of each target gene in inoculated plants relative to the calibrator non-inoculated plants. Asterisks indicate the significance of the 2-ΔCt values from the calibrator (*p* ≤ 0.01; *p* ≤ 0.001; *p* ≤ 0,0001; Student’s *t*-test)

A qPCR assay was performed at three time points (0, 7 and 15 DPI) on 14 target genes that resulted differentially expressed in infected and non-infected roots of the two analyzed genotypes. The aim of this assay was to monitor the expression of key genes identified in previous microarray experiments belonging to major gene categories involved in plant defence response. A distinct gene expression pattern between the two genotypes in the *inoculated vs not inoculated* conditions was evidenced. At time point 0 (Fig. 2 panel a) the majority of the analyzed genes resulted down-regulated, except for Phosphatase and Jasmonate ZIM domain protein genes in the resistant line, a Beta-1,3-glucanase and a Peroxidase4 in the susceptible line and a WRKY transcription factor up-regulated in both varieties. Almost all the target genes resulted up-regulated in both genotypes at 7 DPI (Fig. 2 panel b), except for the Acidic Chitinase, under-expressed in the resistant line. A strong response in both genotypes to the FORL challenge was evidenced, particularly for genes directly involved in the resistance process, significantly up-regulated at this time point. At 15 DPI, an up-regulation of CC-NBS-LRR resistance protein, CYP83B1 cytochrome p450, Dehydrin, Phosphatase and WRKY transcription factor was observed in the incompatible interaction, confirming results obtained in the microarray experiment (Fig. 2 panel c). As for the compatible interaction, most of the target genes resulted up-regulated in last two qPCR experiment timing points.

### Gene enrichment analysis

**Fig. 3:**
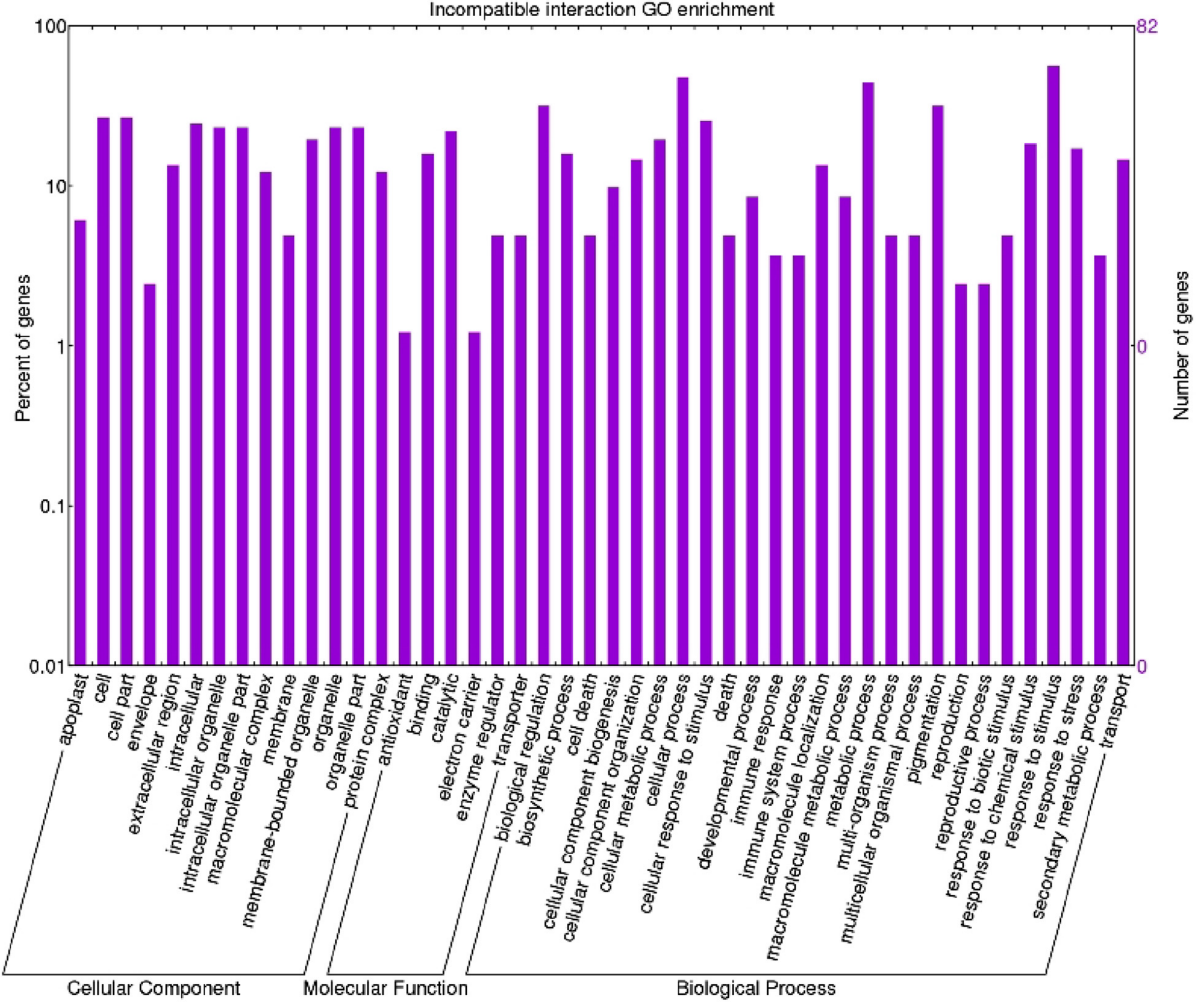
Enriched GO term distribution of the incompatible interaction. Functional analysis of the differentially expressed genes in the *Momor*-FORL interaction 15 days post inoculum. The Y-axis indicates the percentage and number of tomato genes in each Gene Ontology (GO) category. X-axis indicates GO categories (Cellular Component; Molecular Function; Biological Process)

**Fig. 4:**
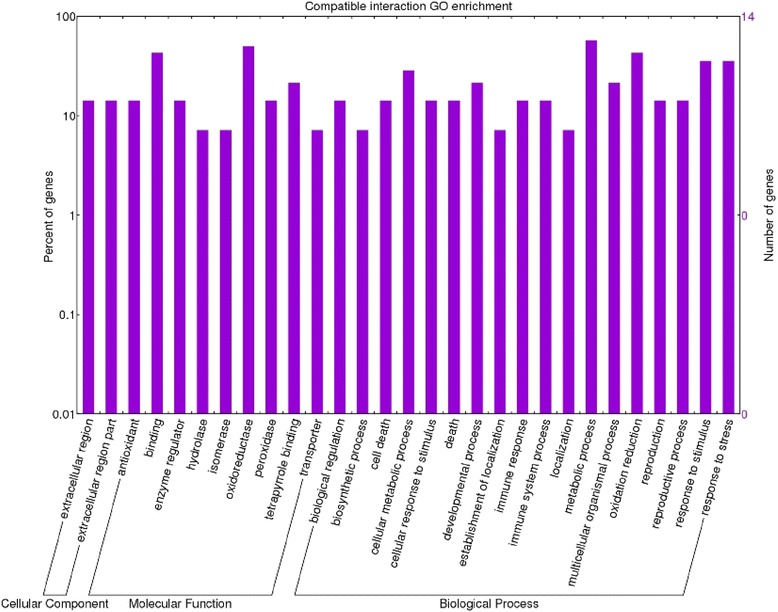
Enriched GO term distribution of the compatible interaction. Functional analysis of the differentially expressed genes in the *Monalbo*-FORL interaction 15 days post inoculum. The Y-axis indicates the percentage and number of tomato genes in each Gene Ontology (GO) category. X-axis indicates GO categories (Cellular Component; Molecular Function; Biological Process)

**Fig. 5:**
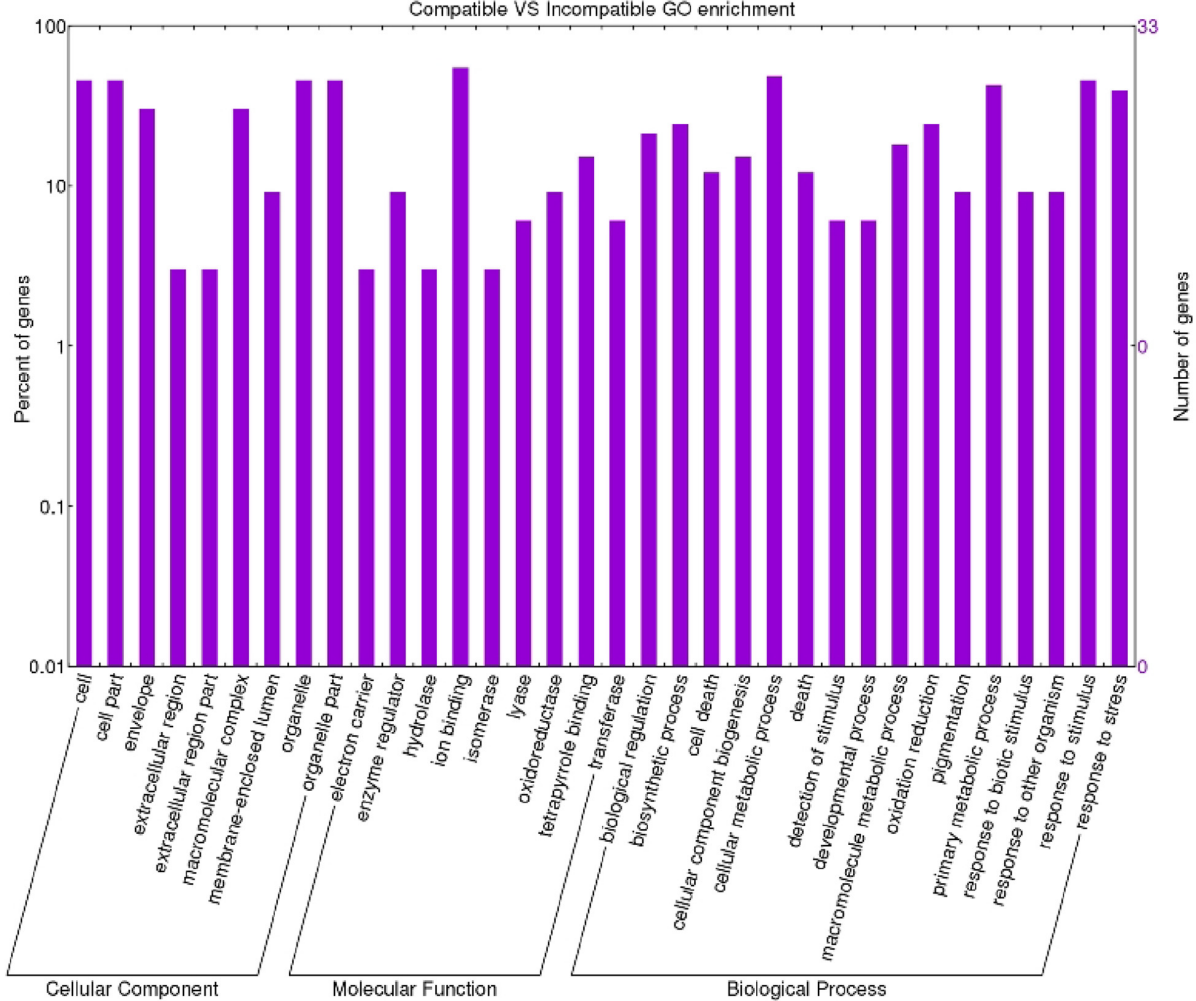
Enriched GO term distribution of the comparison between compatible and incompatible interactions. Functional analysis of the differentially expressed genes in the comparison between *Momor* and *Monalbo* genotypes inoculated with FORL. The Y-axis indicates the percentage and number of tomato genes in each Gene Ontology (GO) category. X-axis indicates GO categories (Cellular Component; Molecular Function; Biological Process)

A GO (Gene Ontology) term annotation analysis was performed of all transcripts identified. Through this analysis we were able to assign functional annotation to the differentially expressed transcripts. Gene ontology analysis performed on the incompatible interaction dataset allowed us to identify 93 enriched functional groups and 68 enriched categories in the compatible interaction dataset. On directly comparing the datasets of the two inoculated genotypes, 198 enriched GO terms were observed. Within the biological process, molecular function and cellular component categories, in the incompatible interaction, the terms ‘metabolic process’, ‘synthase activity’, ‘synthase complex’, ‘biosynthetic process’ and ‘response to’ were dominant (Fig. 3). In particular, seven specific GO terms associated with the synthesis of glucosinolates were found (‘indoleglucosinolate biosynthetic process’, GO:0009759 –‘S-glycoside biosynthetic process’, GO:0016144 –‘glucosinolate biosynthetic process’, GO:0019761 –‘glycosinolate biosynthetic process’, GO:0019758 –‘S-glycoside metabolic process’, GO:0016143 –‘glucosinolate metabolic process’, GO:0019760 –‘glycosinolate metabolic process’, GO:0019757). This finding allowed us to consider glucosinolates as well as tryptophan-derived metabolites as major players in tomato FORL resistance. Interestingly, the cytochrome p450 gene ‘Solyc09g092640.2.1’, involved in the tryptophan metabolism, is present in the above mentioned GO categories as well as in the ‘cell wall modification’ (GO:0042545). Several other enriched GO terms correlated with changes in cell wall structure were found in this interaction: ‘cell wall thickening’ (GO:0052386) and ‘callose deposition in cell wall’ (GO:0052543); ‘cellular macromolecule localization’ (GO:0033036 – GO:0070727); ‘callose deposition in phloem sieve plate’ (GO:0080165), ‘polysaccharide localization’ (GO:0033037) and ‘callose localization’(GO:0052545); ‘vascular and phloem transport’ (GO:0010233). Enriched GO categories involved in signal transduction, transcription factor activation and cellular response to stimulus (GO:0007165 – GO:0009719 – GO:0051716 – GO:0060416 – GO:0071495 – GO:0009628) were also detected, whereas in the compatible interaction the terms ‘oxidation process, metabolic process, cell death’, ‘oxidoreductase activity, antioxidant activity and binding’, ‘extracellular’ were the most abundant for the biological process, molecular function and cellular component, respectively (Fig. 4). In the compatible versus incompatible dataset (Fig. 5) different GO terms regarding response to stimulus and metabolic process were detected, suggesting an intense action of response to the pathogen. In particular, GO terms regarding the metabolic process were investigated further since they revealed interesting activation of pathogenesis-related proteins involved in plant-pathogen interactions.

### Model of tomato–FORL incompatible interaction

Transcriptional profile investigation and GO term enrichment analysis were used to reconstruct pathways involved in tomato-FORL interaction during an incompatible response. The incompatible interaction revealed changes especially in signal transduction, metabolic process, tryptophan metabolism and cell wall modifications. Interestingly, the cytochrome p450 gene (Solyc09g092640.2.1) was present in several enriched GO categories related to production of glucosinolates and tryptophan-derived metabolites and to cell wall modifications. The involvement of this gene in such metabolic pathways, activated during pathogen responses, let us to suppose that it has an important role in the resistance process. It is worth noting that ‘Solyc07g056260.2.1’, a glucan synthase also known as callose synthase 7, was overrepresented in all GO term categories related to cell wall structure changes. GO categories involved in cellular response to stimulus, signal transduction and transcription factor activation were also enriched in this interaction.

**Fig. 6:**
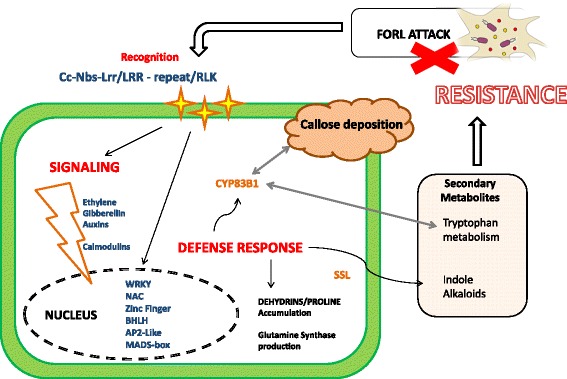
Incompatible interaction model. Graphical representation of *Momor* -FORL interaction at cellular level. Up-regulated resistance proteins are represented with yellow stars and important steps of the reactions in red. Up-regulated DEGs are in blue. Enzymes involved in the defense response are in orange

Combining the results obtained we were able to outline a model of tomato-FORL incompatible interaction (Fig. 6). The presence of up-regulated CC-NBS-LRR, LRR-repeat and RLK resistance proteins suggests an active pathogen recognition, leading to a signaling cascade mediated by hormones like ethylene and especially calmodulins. This signaling cascade activates several families of transcription factors, triggering a double level defense response: activation of CYP83B1 and SSL (Strictosidine synthase-like) genes. The first is involved in the production of tryptophan-derived secondary metabolites against the pathogen and the deposition of callose onto the cellular membrane. The SSL gene could lead to the production of indole alkaloids as secondary metabolites that have a negative effect on the pathogen attack. At the same time, the up-regulation of GST (Glutathione S-Transferase) genes supports the hypothesis of some mechanism of plant detoxification from all the secondary metabolites, that in larger amounts could be negative for the plant itself. Finally dehydrin could act as a regulator of the cell osmotic potential maintenance after FORL root challenge.

### Model of tomato-FORL compatible interaction

**Fig. 7:**
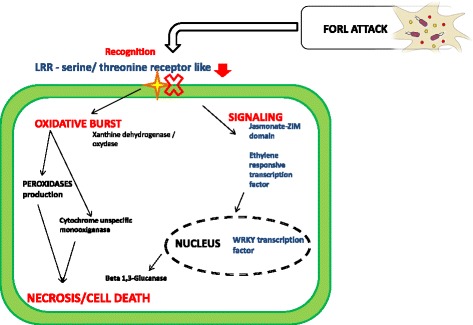
Compatible interaction model. Graphical representation of *Monalbo*-FORL interaction at cellular level. Down-regulated LRR resistance protein is represented with yellow stars “blocked” by a red cross and the major steps of the reactions are in red. Up-regulated DEGs are in blue

The compatible interaction showed a totally different reaction to the pathogen challenge. Oxidoreductase activity seems to play a central role in this interaction since different enriched GO terms associated with this kind of molecular function were found (‘oxidoreductase activity, acting on paired donors, with incorporation or reduction of molecular oxygen’ GO:0016705 –‘oxidoreductase activity’ – GO:0016491 ‘response to oxidative stress’, GO:0006979 – ‘superoxide metabolic process’, GO:0006801). Among these, we detected xanthine dehydrogenases and haem peroxidases, usually involved in the plant biosynthesis of the cell wall, defense responses to wounding and in the oxidative polymerization of lignin subunits as well as increased production of ROS and synthesis of secondary metabolites. Interestingly, a cytochrome p450 (Solyc10g080840.1.1) also seems to be involved in this interaction. This monooxygenase acts on a great variety of substrates: reactions catalyzed include hydroxylation, epoxidation, N-oxidation, sulfoxidation, etc. Host programmed cell death induced by symbiont (GO:0034050), plant-type hypersensitive response (GO:0009626) and a clear up-regulation of cellulase activity (Beta-1 3-glucanase), was evidenced in *Monalbo*-FORL interaction. Such findings could be correlated to the necrosis reaction visually assessed in susceptible plants. Indeed, comparing the results between the two inoculated genotypes (experiment 3) enriched categories were found in the susceptible sample involved in pathogenesis (Solyc01g008620.2.1 Beta-1 3-Glucanase; Solyc03g098740.1.1 Kunitz trypsin inhibitor; Solyc05g050130.2.1 Acidic Chitinase; Solyc11g021060.1.1 Proteinase inhibitor; Solyc03g020060.2.1 Proteinase inhibitor II) and other interesting GO terms related to response to stress (GO:0006950), defense response to fungus (GO:0050832), detection of biotic stimulus (GO:0009595) and cell death (GO:0008219). Such findings led us to postulate a totally different model of interaction between tomato and FORL: *Monalbo* seems to exhibit a much weaker and slower response to the pathogen compared to the resistant genotype. First, during the recognition phase, there is a down-regulation of membrane receptor, LRR-serine/threonine protein kinase. Secondly, the reaction continues directly with the activation of an oxidative burst mediated by peroxidases and a cytochrome monoxygenase. Thirdly, ethylene and jasmonate signaling molecules activate a signaling cascade that induces transcriptional triggering, mediated by a WRKY transcription factor, leading to a cellular necrosis reaction. This occurrence is supported not only by the presence of an up-regulated Beta1 3-glucanase, an enzyme involved in degradation of the cell wall, but also by the enzyme activities of the initial oxidative burst (Fig. 7).

## Discussion

A global transcriptomic profile of tomato-FORL interaction was performed through four different experiments for assessing transcripts activated or inhibited during the resistant and the susceptible reaction. The tomato–FORL interaction seems to follow the typical reaction of necrotrophic pathogens, activating receptors that recognize pathogen-derived proteins and inducing the production and transport of three major defense hormones, namely SA, JA and ET (respectively Salicylic Acid, Jasmonate, Ethylene) [22–24]. In the incompatible interaction, cellular signaling cascades and regulation of numerous target proteins involved in plant growth, development and defense response, through transcriptional and/or post-translational activation of transcription factors, lead to the induction of plant defense genes [25–27]. In particular, calmodulins/calcium sensor proteins and calmodulin-related proteins seem to play an active role in tomato-FORL interaction. This finding indicated that the resistant genotype is more capable of deploying a wide variety of defense responses for preventing pathogen colonization. Furthermore, incompatible reaction GO category enrichment analysis showed that tryptophan metabolism/biosynthesis and callose deposition in the cell wall play a key role in the response to FORL. CYP83B1, a monooxygenase involved in tryptophan, and especially glucosinolate metabolism, is over-expressed during this interaction. Glucosinolates and their products have a fungistatic effect on *Fusarium spp.* [28, 29] and hydrolysis of its products also influences responses of biotrophic pathogens [30]. Moreover, high production of tryptophan-derived metabolites was observed in tomatoes resistant to tomato yellow leaf curl virus [31]. CYP83B1 is also involved in cell wall modifications and callose deposition, together with the callose synthase7 enzyme. Callose can strongly combat penetration of soil-borne fungi when deposited in elevated amounts [32]. The presence of an up-regulated strictosidine synthase-like (*SSL*) gene supports the hypothesis that monoterpenoid indole alkaloids could be released during this interaction. This enzyme, localized to the epidermis of the apical meristem of roots [33], catalyzes the initial step of monoterpenoid indole alkaloids (MIAs) pathway by condensing the tryptamine, synthesized from tryptophan, with the monoterpenoid secologanin, producing strictosidine, a common precursor of a wide range of different MIAs [34]. Expression of this gene can be induced by ethylene AP2/ERF-domain transcription factor (Solyc03g044300.2.1), up-regulated in our experiment and already proved to be involved in activating plant defense responses [35]. Strictosidine synthase-like proteins have also been identified during plant defense activated against pathogens such as the *Cucumber mosaic virus* and *Alternaria brassicicola* [36]. The cellular damage induced by the necrotrophic pathogen could also lead to water loss [37], and the activation of a dehydrin (Solyc12g099390.1.1) and a glutamine synthetase (Solyc04g014510.2.1) in the resistant genotype could help to redress the osmotic stress, avoiding FORL-induced root and crown rot [4] evidenced a high level accumulation of dehydrin proteins in *Momor* plants infected by FORL, as well as larger amounts of glutamine synthetase (EC: 6.3.1.2; Solyc04g014510.2.1), an enzyme involved in the nitrogen assimilation pathway, supporting our results. Glutamine synthetase could alter glutamate metabolism, resulting in an “endurance” state as already reported in other necrotrophic pathogen interactions [38]. Endurance can be defined as a state in which cell viability is maintained via nitrogen (N) reutilization and involved in a senescence-natured ‘slash-and-burn’ defense response [39]. Translocation of N toward the invaded area proved to be effective for a resisting host [40, 41]. The up-regulation of a glutathione S-transferase (GST), together with the increased protein levels found in the *Momor*-FORL interaction [4], supports its involvement in the resistance process. Since the *Momor* genotype constitutively showed higher amounts of glutathione S-transferase regardless of FORL infection, it could be inferred that this protein is involved in the resistance process [4]. It is well known that GST contributes to mitigate further oxidative damage in cells surrounding the infected areas [20, 42, 43].

In the compatible interaction an up-regulated Jasmonate ZIM-domain protein and an up-regulated Omega-6 fatty acid desaturase were detected. Generally, JA and ET play an important role in defense responses to necrotrophic pathogens and chewing insects, while SA is more involved in responses to biotrophs and sucking insects [44, 45]. Investigation of the tomato-FORL interaction at proteomic level confirms the presence of higher amounts of peroxidases in the compatible interaction [4]. An unspecific monooxygenase could also be involved in oxidoreductase activity and in necrosis in the susceptible variety in response to a pathogen. GO terms correlated with the metabolic process and response to stress, including several genes coding for PR-proteins like Beta1,3-glucanase, chitinases and protease inhibitor, were up-regulated in the comparison between the two inoculated genotypes. PR-proteins accumulate locally in the infected and surrounding tissues and also in remote uninfected tissues [46]. Among these proteins Beta1, 3-glucanases and chitinases are very abundant hydrolytic enzymes in plants infected by fungi and play a major role in defense reactions against fungal pathogens by degrading the cell wall [47]. The qPCR assay helped us to better depict the tomato-FORL interaction. The distinct gene expression pattern emerging between the two genotypes in the *inoculated vs not inoculated* conditions revealed that at 0 DPI, the great majority of genes was down-regulated for both genotypes, with the exception of WRKY transcription factor involved in the early stages of signaling and activation of defense response in plants. At this stage another signaling protein (a Phosphatase) was up-regulated in the resistant line, suggesting that in such genotype the alert components are induced very rapidly. The resistant genotype is clearly more capable to activate signaling component for preventing the pathogen colonization and simultaneously to compensate the overall stress induced by the pathogen through the up-regulation of genes involved in both osmotic potential maintenance (dehydration-induced proteins) and cellular detoxification (Glutathione-S-transferase). The susceptible genotype shows a totally different response to the pathogen, characterized by the pronounced activation of an oxidative burst that induces cells to degeneration and necrosis.

## Conclusions

Transcriptome analysis proved to be very useful in recognizing tomato molecular layouts available to fight the pathogen invasion and, furthermore, to elucidate mechanisms of interaction between life forms. The resistant genotype manages the pathogen attack thanks to a key gene (CYP83B1) and maintaining cellular fitness, while the susceptible one tries to alert the plant of pathogen infection activating its defense arsenal but it fails because lacks the resistance machinery. Our work allowed more deep understanding of the molecular basis of the tomato-FORL interaction and, furthermore, could be considered as a starting point both for future functional studies and the improvement in disease control strategies.

## Availability of data and materials

The microarray datasets supporting the results of this article are available at the NCBI’s GEO dataset (http://www.ncbi.nlm.nih.gov/gds) under the series ID GSE71393.

## Additional files

Additional file 1: Table S1. Primer sequences used for qPCR assay; **Table S2.** Microarray experiment design. (DOCX 14 kb) Additional file 2: Figure S1. RT qPCR assay of *Pal (Phenylalanine ammonia lyase) (a) Catalase (b), Beta-Glucosidase (c), Receptor-like protein kinase (RLK)4 Serine/Threonine (d)* genes on *Marmande* variety infected and not infected roots to monitor the FORL infection. Bars indicate the RQ (relative quantity) of target genes in the inoculated and control conditions. Error bars represent standard deviations calculated for the qPCR results of three biological replicates. (PPTX 621 kb)

## Abbreviations

DE genes: differentially expressed genes
DPI: days post inoculum
ET: ethylene
FDR: false discovery rate
FHB: *Fusarium* head blight
FOC: *Fusarium oxysporum f.sp. cubense*
FON: *Fusarium oxysporum f.sp. niveum*
FORL: *Fusarium oxysporum f.sp. radicis-lycopersici*
GO: gene ontology
GST: glutathione S-transferase
JA: jasmonate
MIAs: monoterpenoid indole alkaloids
NGS: next generation sequencing
PAL: phenylalanine ammonia lyase
RLK: receptors like kinases
SA: salicylic acid
SSL: strictosidine synthase-like
TCs: tentative consensus sequences
