# Supplementary material for: Fusarium oxysporum f.sp. radicis-lycopersici induces distinct transcriptome reprogramming in resistant and susceptible isogenic tomato lines
Source: BMC Plant Biol. 2016 Feb 27;16:53. doi: 10.1186/s12870-016-0740-5 (PMC4769521; doi:10.1186/s12870-016-0740-5)
Supplement: Additional file 1: Table S1. — Primer sequences used for qPCR assay; Table S2. Microarray experiment design. (DOCX 14 kb) [file 12870_2016_740_MOESM1_ESM.docx]

|  | **Target gene** | **Forward primer (5’-3’)** | **Reverse primer (3’-5’)** |
| --- | --- | --- | --- |
| Solyc09g065820.2.1 | *BHLH transcription factor* | GTCGAAAACAGCTCCGATCC | CCGCCAAATTAGGTTCTGCT |
| Solyc10g079860.1.1 | *Beta-1,3-glucanase* | TTTCCAATTACCGCGACGAC | TAGAGCCACAACATCTGCTG |
| Solyc07g005380.2.1 | *PR-10 (Pathogenesis related)* | ATTCTGCGTCCATCACATCC | GTTCCATAGAGCTCCCATGC |
| Solyc11g011030.1.1 | *Pto-responsive gene* | GCTGAGCTTATAATTGACCTGGT | TGTGGATGCATTTGAAGGGTC |
| Solyc05g050130.2.1 | *Acidic Chitinase* | TCCTAGTGTTGGTGGATGCA | AACTTCCAACACCACCACCA |
| Solyc04g015210.2.1 | *CC-NBS-LRR* | TCCGTTCGTCAAGTGGAACA | TGGGTAGTTTCTCCAGGCTG |
| Solyc09g092640.2.1 | *CYP83B1 (Cytochrome p450)* | TATCTCAACAAGCTGCGTGT | TGCGCTTCTTCATCAAACCT |
| Solyc12g099390.1.1 | *DEHYDRATION-INDUCED protein (Dehydrin)* | AGGGTGAAGAGGAGTCAAGG | GCCTCAATAGCATGCTCTTCA |
| Solyc03g121420.2.1 | *Phosphatase* | CCACACAAATCCAGGCTACG | ACATGTTGGGAGGGCAAGTA |
| Solyc08g006320.2.1 | *WRKY transcription factor 3* | TATCGCCGTTGACAAATCCG | CGGAACAGCTTCACATCCAA |
| Solyc12g009220.1.1 | *Jasmonate ZIM-domain protein 1* | TGATCAACCAGAGAAGGCACA | TGTGGGGTTCTGTTTGTTGG |
| Solyc04g071890.2.1 | *Peroxidase 4* | GAAGCTCAAAGGCCTGCTTT | GCTTGGCCAACATCATCCTT |
| Solyc10g080840.1.1 | *Cytochrome P450 (unspecific monooxigenase)* | CGCCACAACGTCTATGGAAG | ACTCTTCTTGTTTTCGCGCA |
| Solyc11g065930.1.1 | *Xanthine dehydrogenase/oxidase* | CTGCCTTGAAGAGAAGCACC | GCAACAGCTCCTTGTTCCAA |

TABLE S1: Primer sequences used for the q-PCR experiment.

TABLE S2: Microarray experimental design

| **EXPERIMENTS** | **GENOTYPES** |
| --- | --- |
| Incompatible interaction | Momor inoculated VS Momor non-inoculated |
| Compatible interaction | Monalbo inoculated VS Monalbo non-inoculated |
| Compatible VS Incompatible | Momor inoculated VS Monalbo inoculated |
| Control reaction | Momor not-inoculated VS Monalbo non-inoculated |
