# Supplementary figures and images for: Fusarium oxysporum f.sp. radicis-lycopersici induces distinct transcriptome reprogramming in resistant and susceptible isogenic tomato lines
Source: BMC Plant Biol. 2016 Feb 27;16:53. doi: 10.1186/s12870-016-0740-5 (PMC4769521; doi:10.1186/s12870-016-0740-5)

## Slide 1
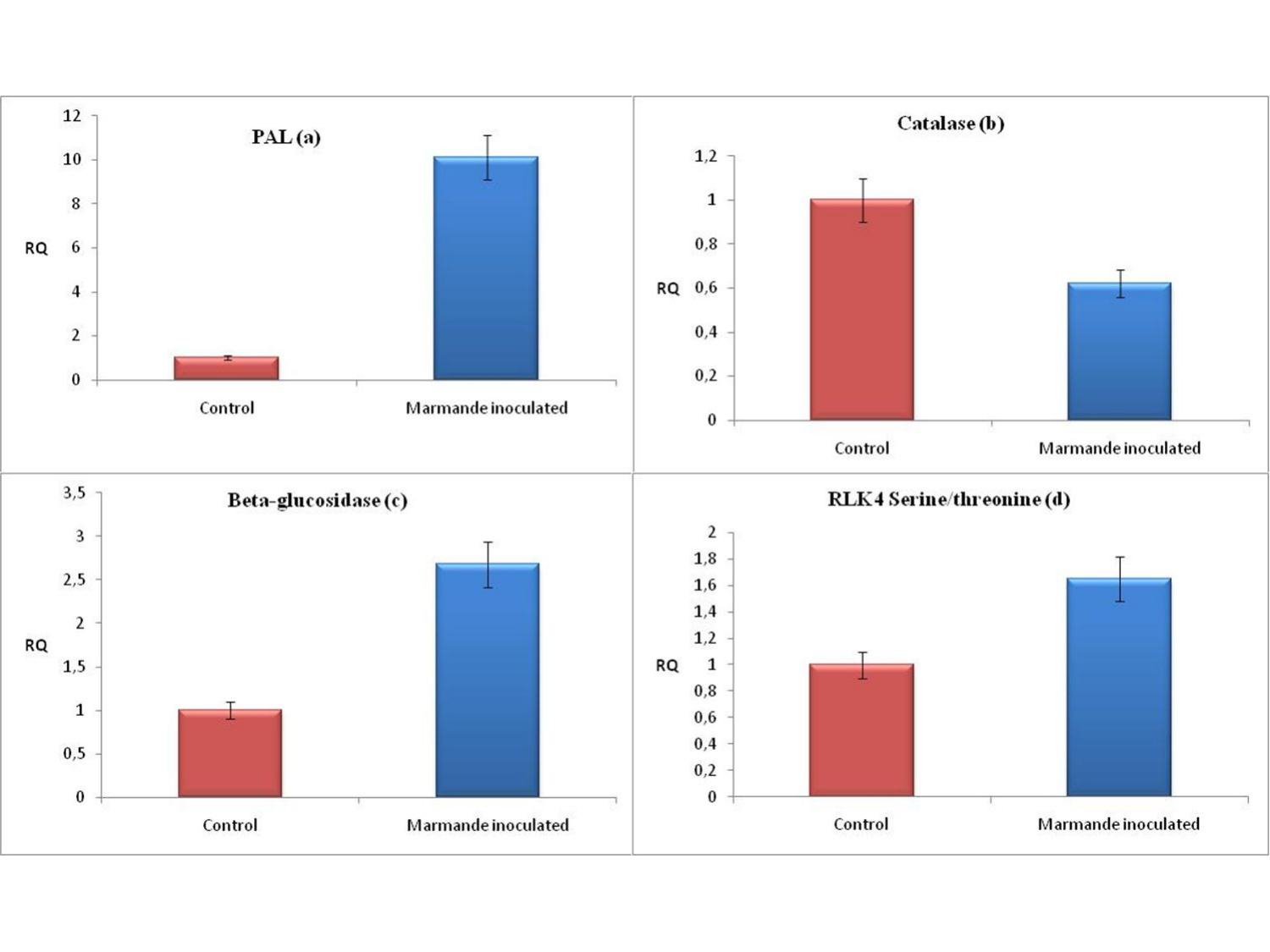

Supplement: Additional file 2: Figure S1. — RT qPCR assay of Pal (Phenylalanine ammonia lyase) (a) Catalase (b), Beta-Glucosidase (c), Receptor-like protein kinase (RLK)4 Serine/Threonine (d) genes on Marmande variety infected and not infected roots to monitor the FORL infection. Bars indicate the RQ (relative quantity) of target genes in the inoculated and control conditions. Error bars represent standard deviations calculated for the qPCR results of three biological replicates. (PPTX 621 kb) [file 12870_2016_740_MOESM2_ESM.pptx]
